# theLiTE™: A Screening Platform to Identify Compounds that Reinforce Tight Junctions

**DOI:** 10.3389/fphar.2021.752787

**Published:** 2022-01-04

**Authors:** Teresa Lopes Gomes, Virgínia de Oliveira-Marques, Richard John Hampson, António Jacinto, Luciana Vieira de Moraes, Rui Gonçalo Martinho

**Affiliations:** ^1^ Thelial Technologies SA, Parque Tecnológico de Cantanhede, Cantanhede, Portugal; ^2^ Thelial BV (Epinutra), Wageningen, Netherlands; ^3^ iNOVA4Health, Chronic Diseases Research Centre - CEDOC, NOVA Medical School, NMS, Universidade Nova de Lisboa, Lisbon, Portugal; ^4^ Departamento de Ciências Biomédicas e Medicina, and Center for Biomedical Research, Universidade do Algarve, Faro, Portugal; ^5^ Department of Medical Sciences and Institute for Biomedicine (iBiMED), University of Aveiro, Aveiro, Portugal

**Keywords:** PAR-6, epithelial cells, tight junctions, quercetin, myricetin, polarity (cell)

## Abstract

Tight junctions (TJ) are formed by transmembrane and intracellular proteins that seal the intercellular space and control selective permeability of epithelia. Integrity of the epithelial barrier is central to tissue homeostasis and barrier dysfunction has been linked to many pathological conditions. TJ support the maintenance of cell polarity through interactions with the Par complex (Cdc42-Par-6-Par-3-aPKC) in which Par-6 is an adaptor and links the proteins of the complex together. Studies have shown that Par-6 overexpression delays the assembly of TJ proteins suggesting that Par-6 negatively regulates TJ assembly. Because restoring barrier integrity is of key therapeutic and prophylactic value, we focus on finding compounds that have epithelial barrier reinforcement properties; we developed a screening platform (*the*LiTE™) to identify compounds that modulate Par-6 expression in follicular epithelial cells from Par-6-GFP *Drosophila melanogaster* egg chambers. Hits identified were then tested whether they improve epithelial barrier function, using measurements of transepithelial electrical resistance (TEER) or dye efflux to evaluate paracellular permeability. We tested 2,400 compounds, found in total 10 hits. Here we present data on six of them: the first four hits allowed us to sequentially build confidence in *the*LiTE™ and two compounds that were shortlisted for further development (myricetin and quercetin). We selected quercetin due to its clinical and scientific validation as a compound that regulates TJ; food supplement formulated on the basis of this discovery is currently undergoing clinical evaluation in gastroesophageal reflux disease (GERD) sufferers.

## Introduction

Epithelial cells are polar—arranged according to an apical-basal axis—and connected by junctional complexes. The apical pole faces the environment whilst the basal pole is anchored to the extracellular matrix. In mammalian epithelial cells, the junctional complex is defined by four structures, from apical to basal: tight junctions (TJ), adherens junctions (AJ), gap junctions and desmosomes. In *Drosophila melanogaster,* the intercellular junctional complex consists of AJ (or zonula adherens) and septate junctions (SJ)—a ladder-like structure functioning as paracellular barrier (Banerjee et al., 2006)—the corresponding to TJ in mammals. TJ are composed of transmembrane proteins: claudins (a protein family with 26 members in human), MARVEL domain proteins (occludin, tricellulin, MARVEL domain-containing protein 3 (MARVELD3)), junctional adhesion molecule (JAM) and membrane peripheral proteins such as zonula occludin (ZO)-1, ZO-2 and ZO-3 (Zihni et al., 2016). TJ seal the intercellular space and form a selective barrier allowing diffusion of substances depending on their size and charge. The integrity of epithelial barriers is crucial for the maintenance of tissue homeostasis; barrier disruption contributes to pathological conditions such as asthma, atopic dermatitis, eosinophilic esophagitis, food allergy, allergic rhinitis, rheumatoid arthritis, systemic lupus erythematosus, type 1 and type 2 diabetes obesity (reviewed in (Akdis 2021) and gastroesophageal reflux disease (GERD) (Orlando and Orlando 2009). Recently, an epithelial barrier hypothesis has been proposed to explain the rise in allergic and autoimmune diseases (Akdis 2021). This hypothesis takes into consideration that environmental changes caused by industrialization, urbanization and westernized life-style can affect the epithelial barrier of the skin, upper and lower airways and gut mucosa. Household cleaning agents, surfactants, enzymes and emulsifiers in processed food have been shown to damage the epithelial barrier and a connection between exposure to these agents and development of asthma, atopic disease, and intestinal permeability has been identified (Akdis 2021).

**FIGURE 1:**
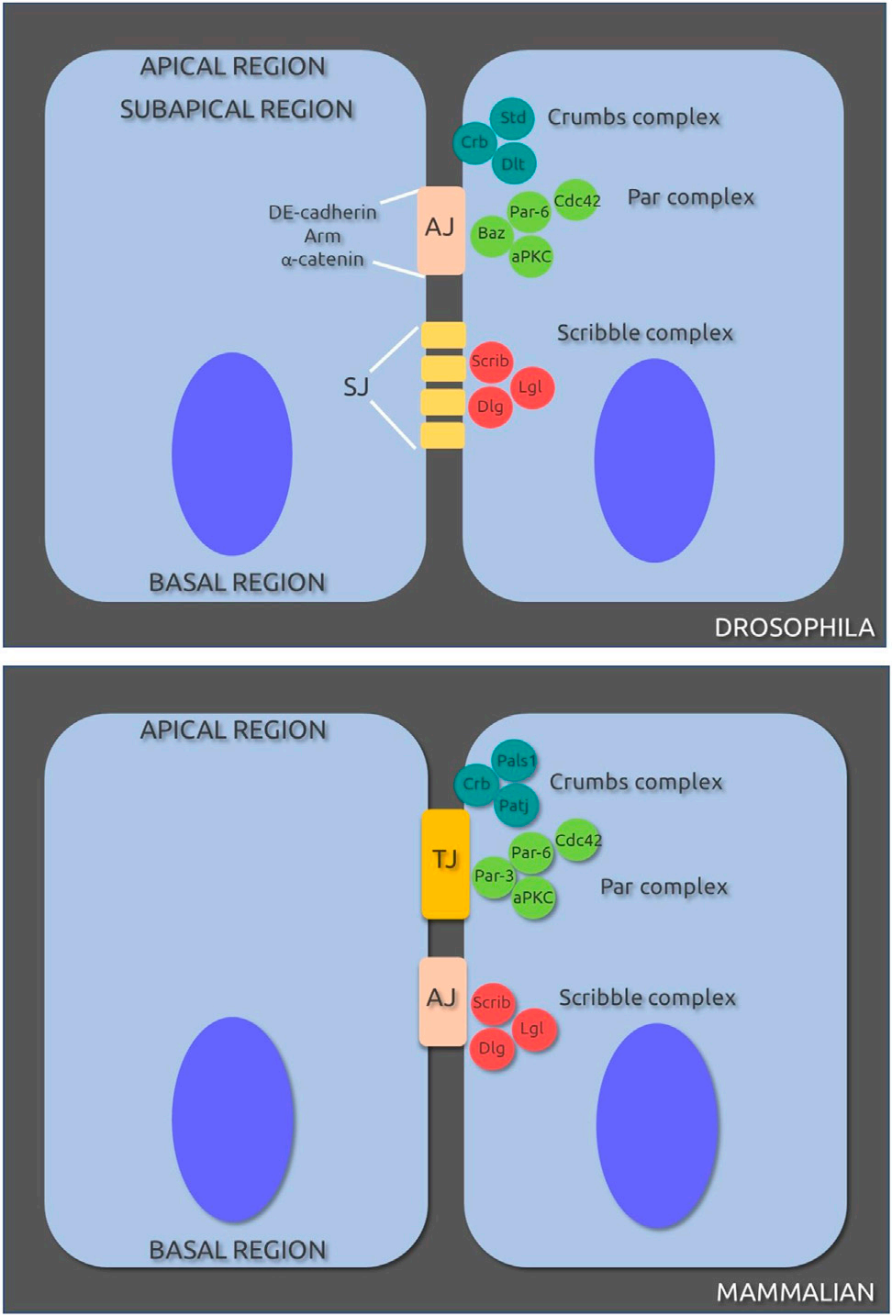
Cell-cell junctions and protein complexes involved in polarity in *Drosophila melanogaster* **(A)** and mammalian **(B)** epithelial cells. AJ, adherens junctions; SJ, septate junctions; TJ, tight junctions. Original sources cited in text; for review see (Assémat et al., 2008).

Cell polarity is essential for correctly functioning epithelial barriers. The maintenance of polarity requires cooperation between components of three different complexes: Protein partitioning defective (Par) complex (Par-6—atypical protein kinase C (aPKC)—Cdc42—Par-3 (Bazooka (Baz) in *Drosophila*) revised in (Joberty et al., 2000; Goldstein and Macara 2007), Crumbs complex (Crumbs (Crb)—Pals1 (Stardust (Std in *Drosophila*)—Paj1 (DiscsLost in *Drosophila*) (Bachmann et al., 2001; Hong et al., 2001; Makarova et al., 2003) and the Scribble complex (Scribble (Scrib)—Lethal giant larvae (Lgl)—Discs Large (Dlg)) (Su et al., 2012) (Figure 1). The interplay between these complexes is also important for regulation of cell polarity and tight junction assembly (Bachmann et al., 2001; Hutterer et al., 2004; Su et al., 2012). Par-6 can interact directly with Pals1 (Hurd et al., 2003) and Lgl (Yamanaka et al., 2003), the Crb and Par complexes are apical membrane determinants and the Scrib complex act as a lateral membrane determinant.

Studies of Joberty et al. (2000) revealed that Par-6 can bind directly to Cdc42, Par-3 and aPKC and that this complex is important in either or both formation and maintenance of normal TJ. Further studies showed that overexpression of Par-6 delays the assembly of TJ proteins (ZO-1, claudin-1 and occludin) in Madin-Darby canine kidney (MDCK) epithelial the Par-6 is a negative regulator of TJ assembly. Based on these findings we developed a compound screening method to target Par-6: Thelial Live Targeted Epithelia (*the*LiTE™) uses egg chambers from *Drosophila melanogaster* (Dm), a model organism with orthologs for over than 75% of human disease genes (Reiter et al., 2001). We monitor the follicular epithelial cells in the DmPar-6-AcGFP in which Par-6 is easily identified in the apical side of the cells; the egg chambers show a Par-6-GFP ring surrounding the chamber clearly visible. These results are consistent with the ones previously observed with a distinct Par6-GFP construct (Wirtz-Peitz et al., 2008; Morais-de-Sá et al., 2010). Our readout is Par-6 ring impairment. Disturbances in the ring pattern after incubation with the tested compound, in a blind fashion, determine its potential candidacy but only if this compound is pharmacologically reliable/suitable. The following step is to confirm barrier reinforcement property of our hit by a standard permeability assay or by research in the literature. Applying a semi-automated format followed by a second manual confirmation, we screened over 2,400 compounds and built a portfolio of six Par-6-targeted candidates using a simple yes/no decision key. Two candidates were prioritized for further development.

Here we describe in full detail our screening assay *the*LITE™ and hypothesize on the potential mechanisms of action underlying Par-6 ring impairment for our six hit compounds. We have advanced with the prioritized compounds and developed a proven safe food supplement supporting GERD sufferers that (at the time of writing this manuscript) is in clinical trial (NL clinical trial registry NL9324).

## Materials and Equipment

### Chemicals

Prestwick Chemical Library (PCL) of 1,200 compounds and Johns Hopkins Clinical Compound Library (JHCCL) containing 1,514 compounds, were used in our screening. For confirmation compounds were re-purchased from separate suppliers. Other reagents were Schneider’s insect medium (Sigma S0146), fetal bovine serum (FBS) (Sigma F4135), Pen/Strep (Sigma P4333), dimethyl sulfoxide (DMSO Sigma-D8418), formaldehyde (Sigma F8775), Dako fluorescence mounting medium (Dako, S3023). Prestwick Chemicals were reconstituted and diluted in DMSO, except for colchicine and y27632 that were prepared in distilled water.

### Equipment

Commercial blender, dissecting microscope (Leica M80), CO_2_ pad (Flypad) attached to a CO_2_ flow regulator (Benchtop Flowbuddy, Genesee Scientific), automatic pipettes (20, 200, 1000 µl) (Zeiss Axiovert 200M microscope attached to a cooled CCD camera (Roper Scientific Coolsnap HQ CCD); images were analyzed using Metamorph 7.7.9.0 and ImageJ software.

### Biological Materials

*Drosophila melanogaster* *(Dm)Par-6-AcGFP* Pin/Cy0 adult males and one–3-day old virgin females were used. The *DmPar-6-AcGFP* was generated based on the construct previously described (Petronczki and Knoblich 2001; Wirtz-Peitz et al., 2008). *DmPar-6* is located in the X chromosome at 16C1. *Dmpar-6-AcGFP* construct was synthetized and inserted into a pUC57 vector (GenScript), with sequence based on endogenous *DmPar-6* gene locus fused with AcGFP1 sequence and flanked by NotI sites: NotI site (1–8)—Promoter region (9–1,108)—5′UTR (1,109–1,430)—DmPar-6 coding sequence + introns (1,431–3,196)—AcGFP1 (3,197—3,913)—Two TAG stops (3,914–3,919)—3′UTR (3,920–5,596)—terminator (5,597–5,946)—NotI site (5,947–5,954). Service for subcloning into pCaSpeR2 was provided by the Cell and Molecular Biology Lab, CEDOC, Portugal; P-element transformation and transformants selection were provided by Thebestgene, retrieving seven lines with *DmPar-6* insertion in different chromosomes. All lines were tested and we used for the screening the line with highest fluorescent signal, with insertion in X chromosome. We use Oregon R as wild-type stock for comparisons. All experiments were conducted in accordance with stipulations by DGAV (Direção Geral de Alimentação e Veterinária) affording the required class 1 level protection for genetically modified organism (GMO). No Institutional approval is required for experiments with *Drosophila melanogaster.*

### Other Materials

Petri dishes for dissection, nylon mesh, yeasted feeding bottles, tips, 96-well black/clear flat bottom plates, plastic tubes (2, 15 and 50 ml), glass recipient for collecting processed flies, glass slides, coverslips.

## Methods

### Experimental Design

Two different operational procedures were applied: automated soaking and manual soaking. The automated operation refers to a medium-throughput analysis designed to screen 48 different compounds in parallel. The manual operation is a low-throughput test, allowing the analysis of a maximum of four different compounds in each experiment. Compounds were first screened by the automated soaking method and the ones that rendered positive results (Par-6 modulation) were confirmed by manual soaking. Screening was done in a blinded-fashion: compound was revealed only after confirmation of its effect on Par-6. Both methods are fully described below.

#### 4.1.1. Automated Soaking

General description: female and male flies were distributed into yeasted feeding bottles (20 flies/test compound, maximum of 48 test compounds) at a ratio of two female:1 male. Flies were kept for 2–3 days; the presence of male flies induces increase in the size of ovaries and facilitates the enrichment of egg chambers. Female flies were transferred to a CO_2_ pad (pad with a constant flow of CO_2_ to keep flies anesthetized), decapitated and processed for the isolation of egg chambers. Egg chambers were distributed in 96-well plates, incubated with test compound for 2 hours at 25°C in the dark and in the absence of movement (no shaking). Egg chambers were fixed and plate was transferred to a fluorescent inverted microscope for the detection of GFP signal. Each step is further described in details and entire procedure is sketched in Figure 1. The set-up is designed to be executed in one standard working day (up-to but not including analysis).

##### 4.1.1.2 Egg Chamber Isolation

Isolation of egg chambers was performed according to methods previously described (Theurkauf et al., 1992). Briefly, anesthetized female flies were transferred to a standard commercial blender containing 250 ml of Schneider’s insect medium supplemented with 0.5% of FBS. Fly structures were disrupted with 2-s pulses performed 3 times, at the lowest speed. Fly homogenate was filtered through a 250 µm nylon mesh to a 500 ml glass beaker. Unprocessed flies were washed-out from the nylon mesh back into the blender to repeat the disruption procedure. Fly homogenates were pooled, egg chambers were left to settle for 5 min and 200 ml of medium was aspirated; the remaining medium-containing egg chambers-was transferred to conical 50 ml tubes. Egg chambers were left to settle for an additional 5 min, medium was removed leaving approximately 1 ml, and fresh supplemented medium was added. The amount of medium to be added depends on the number of compounds to be tested considering that approximately 200 µl of medium-containing egg chambers are required per well (for example, if we are testing 20 compounds (1 compound/well) then 4–5 ml medium should be added). The medium-containing egg chambers was transferred to a Petri dish to increase the dispersion of the eggs and then 100 µl of the medium were added to each well of the 96-well plate in two rounds. After 5 min, 100 µl of medium were removed from wells which further received the test compound in the same volume, 2x concentrated (further detailed below).

##### 4.1.1.3 Incubation With Test Compounds and Fixation

Egg chambers were incubated with compounds from PC or JHCC libraries at a final concentration of 30 µM (PC) or 60 µM (JHCC and quercetin) in 0.6% DMSO in incubation medium (Schneider’s medium, 10% FBS, 0.5% Pen/Strep) or in DMSO 0.6% only. The referred concentration was chosen to take a snapshot at a meaningful dynamic range. Positive results were tested for doses 10-fold and 100-fold diluted. More specifically, all compounds were reconstituted in DMSO at 5 mM (stock solution). For screening, compounds were diluted to 60 µM or 120 µM in incubation medium (2x concentrated; v/v) and 100 µl were added to each well. Egg chambers were incubated for 2 h with test compound at 25°C in the dark without shaking. After incubation period, 100 µl of medium was removed and 200 µl of fresh Schneider’s medium was added to wash the egg chambers. After carefully removing 200 µl of the well content, 100 µl of 7.3% of PFA (2-fold concentrated) was added; egg chambers were incubated for 15 min at room temperature (RT). PFA was then removed, followed by two washes of 200 µl with 1X PBS. One hundred microliters of PBS containing 4% anti-fading agent Dabco was added. Plates were kept at 4°C in the dark until analysis, for a maximum of 4 days.

#### 4.1.2. Manual Soaking

**FIGURE 2:**
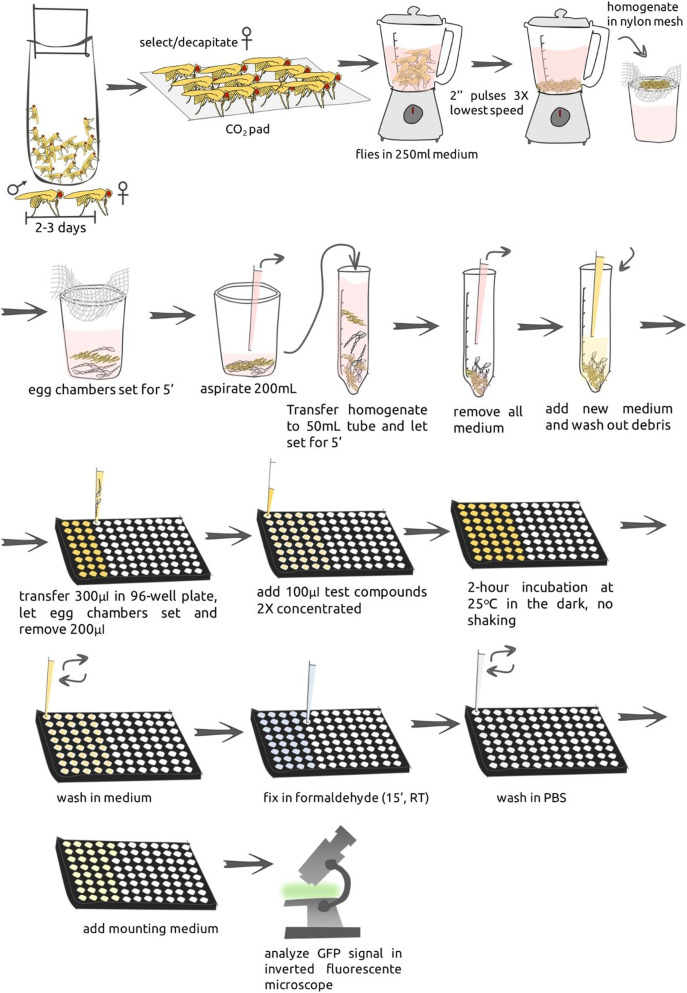
Sequence of steps for automated soaking protocol. Female and male flies are put together into yeasted feeding bottles (2 female:1 male) for 2–3 days. Female flies are transferred to a CO_2_ pad, decapitated and processed using a blender for the isolation of egg chambers. Egg chambers are then distributed in 96-well plates, incubated with test compound for 2 hours at 25°C in the dark, without shaking. Egg chambers are washed, fixed in PFA for 15 min at RT in the dark. Fixation solution is removed and anti-fading Dabco solution added, and plate transferred to a fluorescent inverted microscope for the detection of GFP signal.

General description: female and male flies were distributed into yeasted feeding bottles (5 flies/test compound, maximum of four different test compounds) at a ratio of two females/1 male. Flies were kept together for 2–3 days. Female flies were transferred to a CO_2_ pad (pad with a constant flow of CO_2_ to keep flies anesthetized), decapitated and processed for the isolation of egg chambers. Egg chambers were distributed in 0.5 ml tubes, incubated with test compound for 2 hours at 25°C in the dark and without shaking. Egg chambers were fixed and transferred to glass slides for the detection of GFP signal using a fluorescent microscope. Each step is further described in detail and a schematic of the entire procedure in Figure 2. The approximate time spent on this procedure is up-to 4 h, excluding analysis.

##### 4.1.2.1. Egg Chamber Isolation

Female flies were transferred to a CO_2_ pad, decapitated and transferred to Petri dish on ice containing 400 µl of Schneider’s medium. Flies were dissected individually under a dissecting microscope to remove ovaries, carefully separating them from the surrounding muscle sheath. Ovaries were broken down by flushing up and down using a pipette. Egg chambers were transferred to 0.5 ml tubes, and dissection medium was carefully removed.

##### 4.1.2.3. Incubation With Test Compounds and Fixation

To the isolated egg chambers in the tube, 1.5 µl of test compounds was added to reach a final concentration of 30 µM or 60 µM (in the case of myricetin and quercetin) in 0.6% DMSO in incubation medium (Schneider’s medium, 10% FBS, 0.5% Pen/Strep). In samples without test compound, DMSO alone was added to the same final concentration. Egg chambers were incubated for 2 h at 25°C without shaking. After incubation period, medium was removed and samples were washed once in Schneider’s medium. Egg chambers were fixed with 200 µl of 3.65% PFA in medium and incubated for 15 min at RT in the dark. Fixation solution was removed and samples were washed 3X with 1X PBS (10 min/wash). One hundred microliters of PBS containing 4% anti-fading agent Dabco was added to the egg chambers and transferred to glass slides, covered with cover slip and immediately analyzed under fluorescent microscope.

### Analysis of Par6-GFP Signal

**FIGURE 3:**
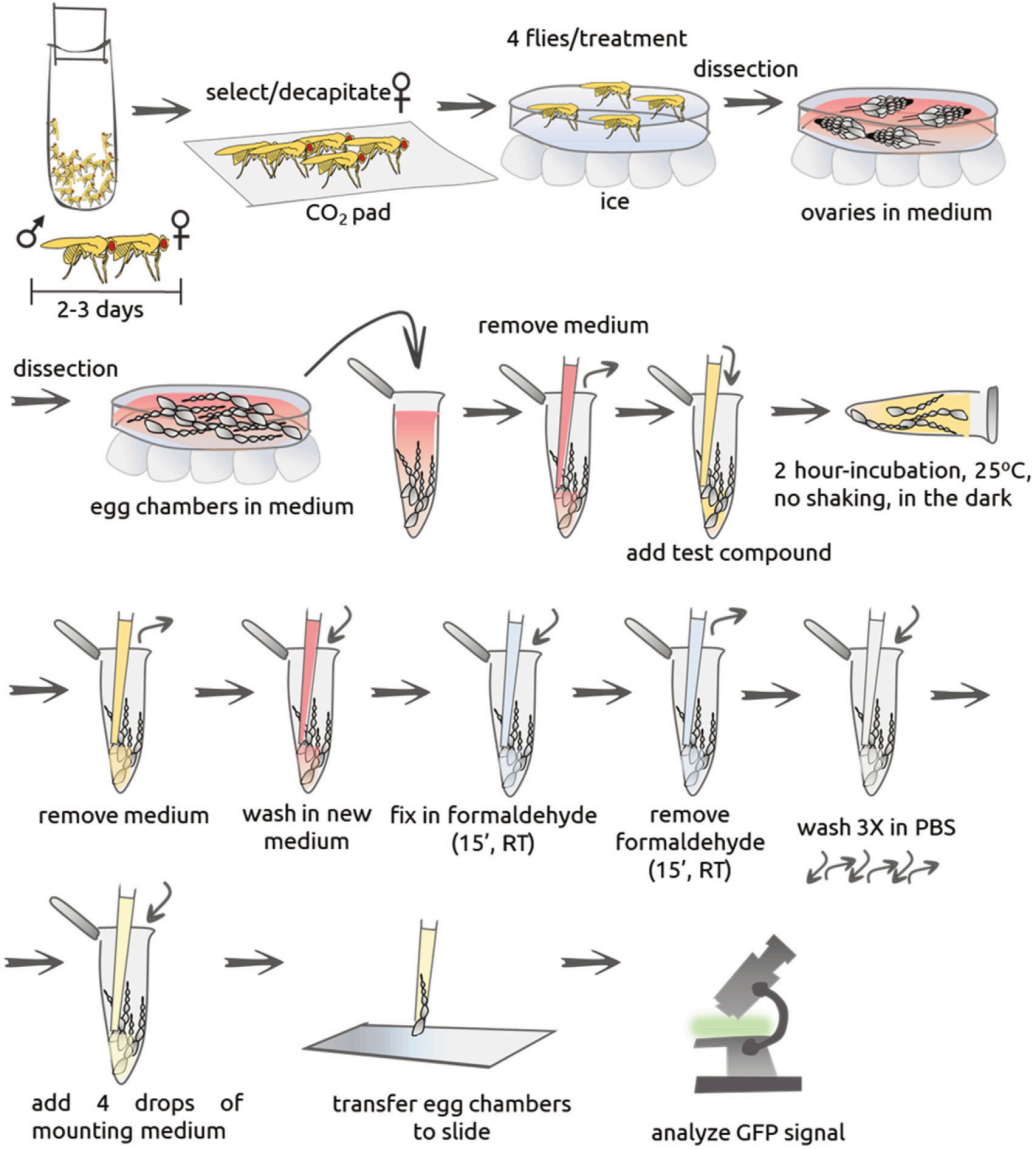
Sequence of steps for manual soaking protocol. Female and male flies are distributed into yeasted feeding bottles at a ratio of two females/1 male, for 2–3 days. Female flies were then transferred to a CO_2_ pad, decapitated and processed for the isolation of egg chambers. The chambers are then distributed in 0.5 ml tubes, incubated with test compound for 2 hours at 25°C in the dark without shaking. Egg chambers are washed, fixed in PFA for 15 min at RT in the dark. Fixation solution is removed and anti-fading Dabco solution added; egg chambers are then transferred to glass slide for the detection of GFP signal using a fluorescent microscope.

**FIGURE 4:**
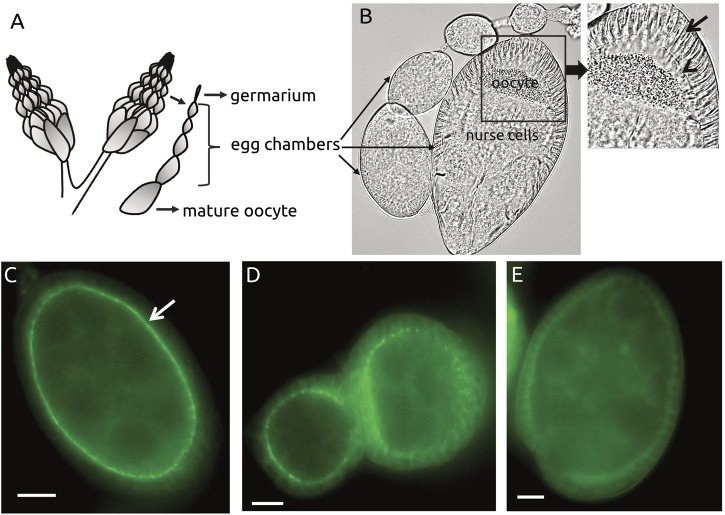
Par-6 ring evaluation in egg chambers. **(A)** Cartoon showing ovaries and egg chambers from *D. melanogaster;* egg chambers mature from apical towards basal. **(B)** Individual egg chamber showing follicular epithelium and nurse cells (nourish the oocyte). Par-6 is localized on the apical side (arrowhead) of epithelial cells (arrow) and evaluated as continuous (white arrow) **(C)** or impaired ring **(D,E)**. Scale bar: 25 µm.

An average of 15–20 egg chambers from stages 6–8 (Figure 3B) were analyzed for each condition, per experiment. The integrity of Par6-GFP signal was evaluated semi-quantitively according to the presence or absence of a continuous Par6 ring (Figure 4). Results were expressed in percentage of egg chambers with impaired or continuous Par-6-GFP ring. Epithelial follicular (surrounding the egg chambers) and nurse cells (the constituents of the egg chamber) were also scored for apparent integrity. Cells with “blebbing” phenotype such as exemplified in Figure 4C, D were considered unviable. Egg chambers with evidence of at least one unhealthy appearing cell (epithelial or nurse cell) were scored as unhealthy. For simplicity we did not distinguish between cell types in presenting the data (number of egg chambers containing unviable cells) as in general, no loss of viability in epithelial cells occurred without detection of unhealthy nurse cells. The window of specific activity of each compound was defined based on the concentration at which a statistically significant percentage of egg chambers with Par-6 ring impairment in the absence of “blebbing” phenotype, all compared to the control (DMSO only).

#### Caco-2 Permeability Assay

To test our results in an industry standard screening we outsourced the confirmation test to a standardized screening provider. Permeability assay using Caco-2 cells (derived from human colorectal carcinoma) was performed by Cyprotex (www.cyprotex.com). In their website the protocol described by (Wang et al., 2000) is referred as their main methodology together with the supplementary information disclosed. Briefly, cells at passage numbers between 40 and 60 were seeded into transwell insert plates (25,000 cells/insert) and cultured for 20 days in Dulbecco’s modified Eagle’s medium supplemented with 10% fetal calf serum, nonessential amino acids (1%, v/v), 2 mM glutamine and penicillin–streptomycin (100 mg/ml) in a humidified atmosphere of 5% CO_2_ at 37°C. The formation of functional epithelial layers was monitored by measuring transepithelial electrical resistance (TEER). Myricetin solution in DMSO was added at three different concentrations (0.3, 3 and 300 µM) to the upper compartment together with Lucifer Yellow (LY) solution for 120 min. The LY solution in the lower chamber was quantified by liquid chromatography with tandem mass spectrometry (LC-MS-MS). The permeability coefficient (P_app_) is calculated with the following equation: P_app_ = (dQ/dt)/(C_0_xA) where dQ/dT refers to the amount of product present in the basal compartment as a function of time; C_0_ is the initial concentration of product applied and A refers to the area of cell monolayer. Results were expressed as 10^–6^ cm/s. TEER was also evaluated at the end of the incubation period; results are expressed as Ωxcm^2^.

#### Clinical Trial With Quercetin

*A clinical trial with quercetin was conducted in GERD* sufferers by researchers at The University of North Carolina at Chapel Hill (Clinicaltrials.gov NCT02226484). Twenty-six patients were recruited under defined eligibility criteria approved by the ethical committee: age (18–80), GERD diagnosis (history of heartburn over 3 times/week for more than 4 months and either abnormal 24 h-pH monitoring or past responsiveness to proton pump inhibitor (PPI) therapy). Other criteria for inclusion were willingness to undergo esophageal biopsy, endoscopy, take study medication and to discontinue or remain off PPIs for the duration of the study. In this open label phase 1 trial patients were given 500 mg quercetin orally (capsules) twice daily ((Pure, House of Nutrition, Yonkers, NY) for 6-weeks. Symptoms were monitored 1 week prior to trial initiation and for the 6-week treatment period. After 6 weeks, endoscopy was carried out and biopsies of the esophageal squamous epithelium (ESE) taken. Changes in the barrier function and acid resistance of ESE were accessed by transepithelial resistance measurements and fluorescein flux. The study is unpublished. Top line data can be disclosed under an agreement between Epinutra (Thelial BV) and The University of North Carolina at Chapel Hill.

### Statistical Analysis

Frequency distribution of the variables were displayed in contingency tables and Fisher’s exact test was applied to establish the statistical significance of the associations between variables. Differences were considered to be significant at *p* < 0.05. Analyses were performed using GraphPad Prism 7.0 software.

## Results

### Par-6 Ring in Fluorescently Tagged Dmpar-6

Fly ovaries are composed of strings of egg chambers at increasingly advanced developmental stages; each egg chamber has germline cells (8 nurse cells and the oocyte) surrounded by follicular epithelial cells (Figures 4A,B). Par-6 is normally expressed at the apical domain of epithelial cells, which in *Drosophila* egg chambers corresponds to the side facing the germline cells (arrowhead in Figure 4B). In fluorescently tagged DmPar-6 this specific localization creates a complete fluorescent ring tracing the apical domain of the epithelial cells (Figure 4C), perfectly distinguishable from wild-type stock (Oregon R (OR)) which lacks significant fluorescent background that could interfere with analysis (Supplementary Figure S1). Figures 4D,E show typical examples of Par-6 ring impairment.

### Live egg Chambers Are Suitable for Chemical Treatments

It has already been shown that isolated egg chambers can be kept in culture for several hours (Prasad and Montell 2007). We then assessed if egg chambers contained unviable cells after incubation with DMSO, the solvent for our chemicals. According to our observations, in the presence of 1% of DMSO and after a 2-h incubation, 92% of egg chambers showed epithelial and nurse cells with a healthy phenotype (total of 24 egg chambers); after 4 h and 30 min 1% DMSO exposure the percentage of egg chambers containing unhealthy cells increased to 76% (total of 25 egg chambers). Importantly, a decrease in Par-6-GFP signal was also observed at the latter time-point (Supplementary Figures S2B,D), compared to the 2 h incubation (Supplementary Figures S2A,C). Based on these results, we established the maximum incubation time of 2 h for all experiments. To overcome the potential effect of DMSO in cell viability which could also impact on Par-6-GFP signal (leading to a false-positive interpretation), we considered that the optimal dose for the tested compounds would be defined by the highest proportion of egg chambers with viable cells that also had Par-6 ring impairment. Therefore, in all experiments we identified the broadest window of specific activity.

### Proof of Principle: Par-6 Protein Is a Suitable Target 

**FIGURE 5:**
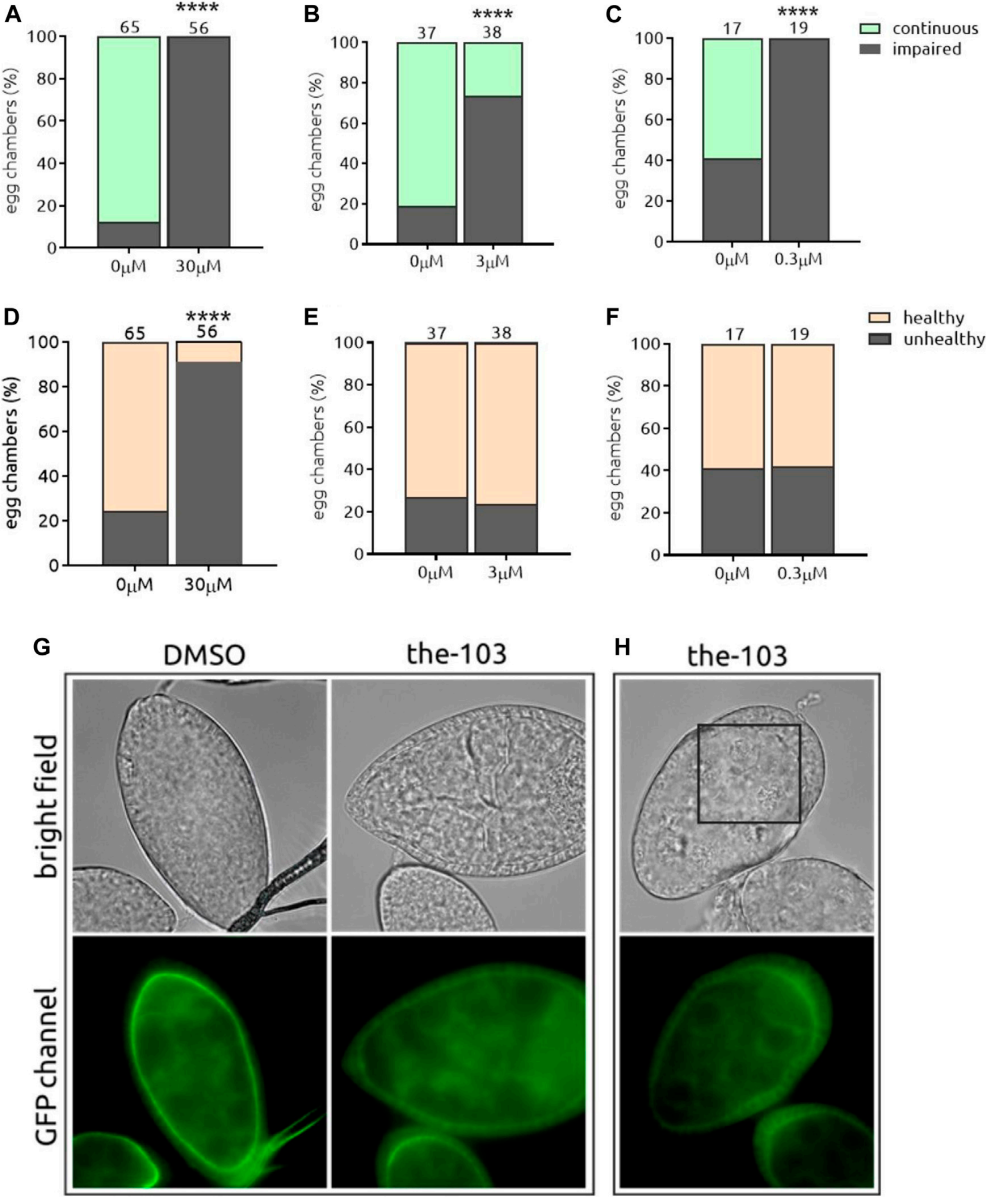
*the*-103 induces Par-6 ring impairment independent of the dose and is toxic at the highest dose. Dissected egg chambers were incubated with three different concentrations of t*he-103* (0.3, 3 and 30 µM) or DMSO only (0 µM) in 0.5 ml tubes for 2 h at 25°C in the dark (no shaking). Egg chambers were washed, fixed in PFA and transferred to glass slide for the detection of GFP signal using a fluorescent microscope and evaluated for Par-6 ring impairment **(A–C)**. Epithelial and nurse cell integrity was evaluated and considered unviable by identifying the presence of a “blebbing-like” phenotype **(D–E)**. Egg chambers containing at least one unviable cell were already considered unhealthy. **(G)** Example of a healthy egg chamber (bright field) incubated with *the*-103 showing discontinuous Par-6 ring (GFP channel). **(H)** Example of an unhealthy egg chamber (bright field) with faded Par-6-GFP signal. Square depicts an area of unhealthy nurse cells. ****p < 0.0001. Scale bar: 25 µm.

We next screened for compounds that could impair Par-6-GFP ring using the PC library in a blinded fashion. We started with the automated soaking protocol (Figure 2), testing up-to 48 different compounds in parallel. Positive results were confirmed individually by manual soaking (Figure 3). We tested in total 2,400 compounds in a blinded-fashion. Here we describe in detail the results of six compounds that impaired Par-6 ring among a total of 10 that yielded positive results (Table 1). Our first hit was Antimycin A (internal code: *the-103*)—a secondary metabolite of *Streptomyces* bacteria (Neft and Farley 1972) and a mitochondrial toxin. Incubation with 30 µM of *the-103* impaired Par-6-GFP ring in all egg chambers tested (Figure 5A) and 91% of them showed evidence of unhealthy cells (Figure 5D) strongly suggesting toxicity. Lowering the dose 10-fold impaired Par-6 ring in 28 of the 38 egg chambers tested (Figure 5B) and when incubated with 0.3 µM all observed egg chambers showed modulation of the Par-6 ring (Figure 5C). *the-103* did not affect cell viability at either of these lower doses (3.0 and 0.3 µM) (Figures 5E,F). For *the-103*, the broadest window of specific activity was established at 3 µM—a dose that induced Par-6 ring impairment in 73% of the egg chambers and at which 76% chambers contained only healthy cells (Table 2). Figure 5 also shows an example of an egg chamber containing Par-6 ring impairment and healthy (Figure 5G) or unhealthy cells (Figure 5H). Because *the-103* can induce mitochondrial dysfunction and cell death (Hytti et al., 2019) we would not consider it as a candidate even at the lowest dose. It is here described as our first positive result and due to its robust effect on Par-6 modulation, *the-103* was thereafter used as a positive control in our assays.

**TABLE 1:** List of the compounds that induced Par-6 ring impairment.

Compound name (CAS#)	Compound code
Antimycin A	the-103
Amphoterracin B	the-104a
Nystatin	the-104 b
Auranofin	the-105
Myricetin	the-110
Quercetin	the-111

**TABLE 2:** Par-6 ring impairment in egg chambers containing healthy cells after incubation with compound. Highlighted is the dose within the window of specific activity.

Compound	Dose (µM)	% Of egg chambers containing	
		Par-6 ring impairment	Par-6 ring impairment and healthy cells
**the-103**	0.0	16.5	94.7
	0.3	100 ****	57.8
	3.0	73.7****	76.3
	30.0	100****	8.9
**the-104a**	0.0	15.4	94.2
	0.3	70.8***	58.4
	3.0	100****	45.9
	30.0	96****	22
**the-104b**	0.0	6.6	95
	0.3	0	100
	3.0	45***	70
	30.0	96.3****	31.7
**the-105**	0.0	6.9	94.8
	0.3	3.8	98.1
	3.0	12.8	95.7
	30.0	58.7****	78.2
**the-110**	0.0	20.7	93.1
	0.6	55.3***	75
	6.0	50.8***	80.7
	60.0	94.5****	76.3
**the-111**	0.0	12.3	98.5
	0.6	45.7**	71.4
	6.0	49.3***	76.7
	60.0	97.6****	40.9

#### 5.3.1. The-104a and The-104 b

**FIGURE 6:**
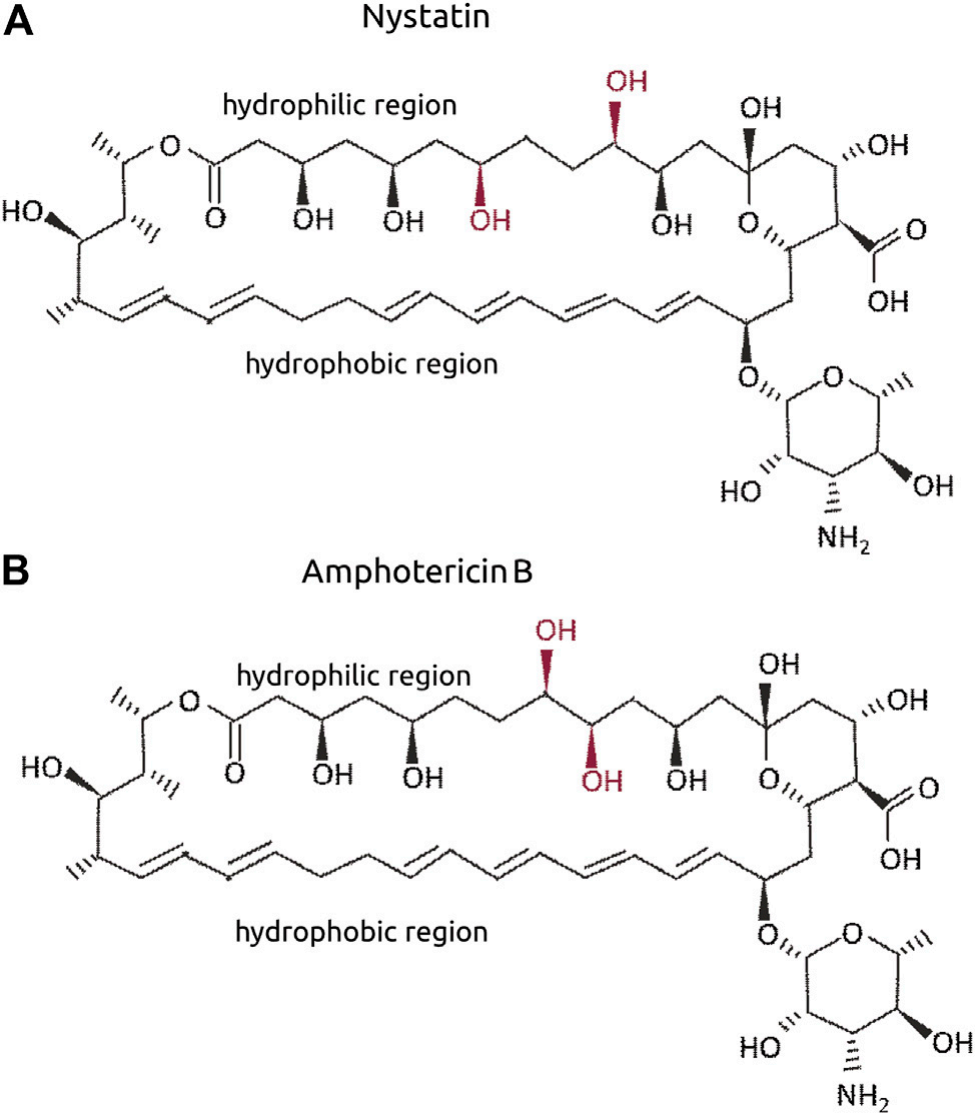
Molecular structure of Nystatin **(A)** and Amphotericin B **(B)**. The OH in red refer to the differential positions when both are compared.

**FIGURE 7:**
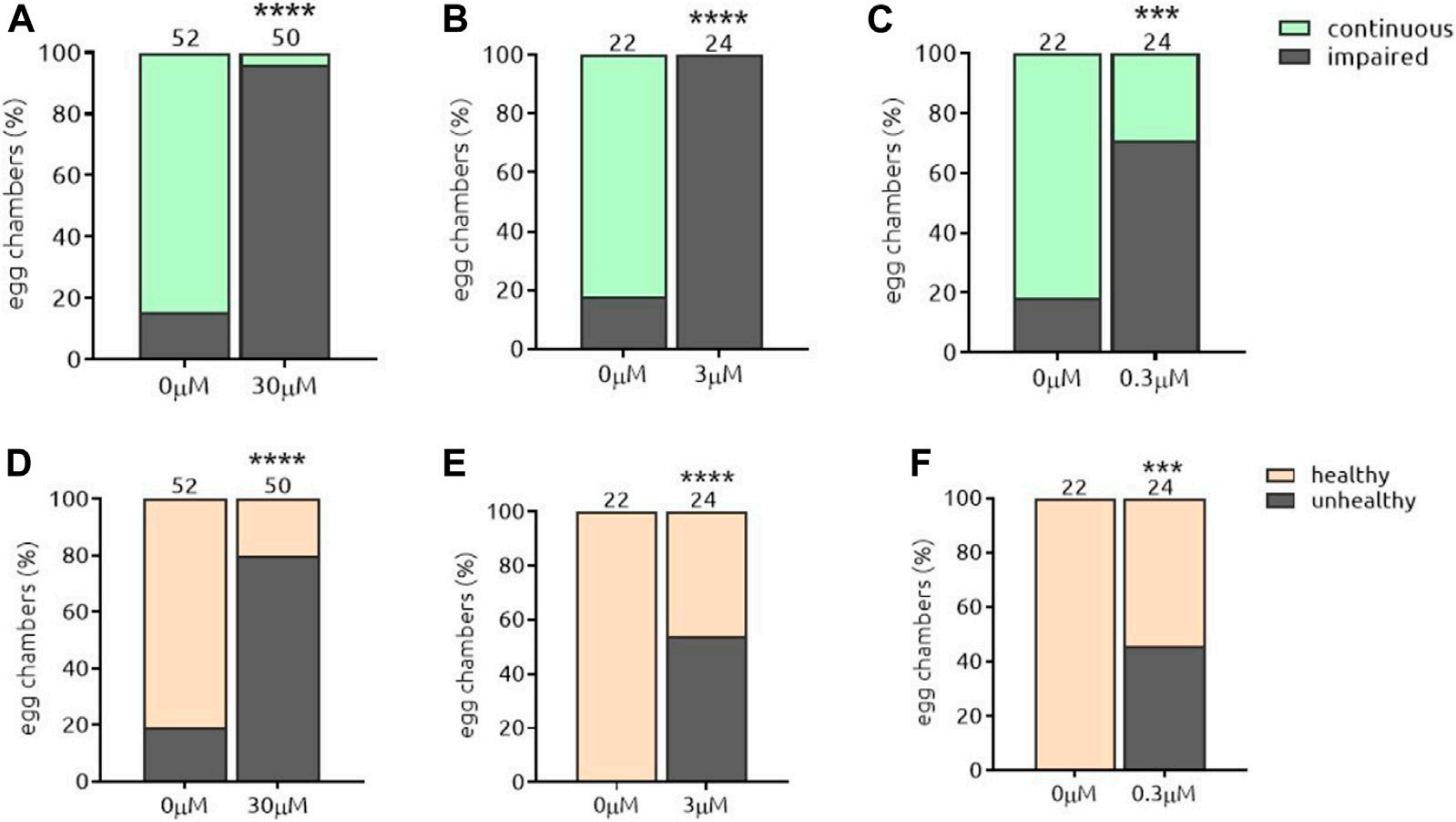
*the*-104a induces Par-6 ring impairment independent of the dose and is toxic at all doses. Dissected egg chambers were incubated with t*he-*104a at 0.3, three or 30 µM or DMSO only (0 µM) in 0.5 ml tubes for 2 h at 25°C in the dark and in the absence of movement (no shaking). Egg chambers were washed, fixed and transferred to glass slide for the detection of GFP signal using a fluorescent microscope for evaluation of Par-6 ring impairment **(A–C)**. Epithelial and nurse cell integrity was evaluated and considered unviable by identifying the presence of a “blebbing-like” phenotype **(D–E)**. Egg chambers containing at least one unviable cell were already considered unhealthy. ***p < 0.001; ****p < 0.0001.

**FIGURE 8:**
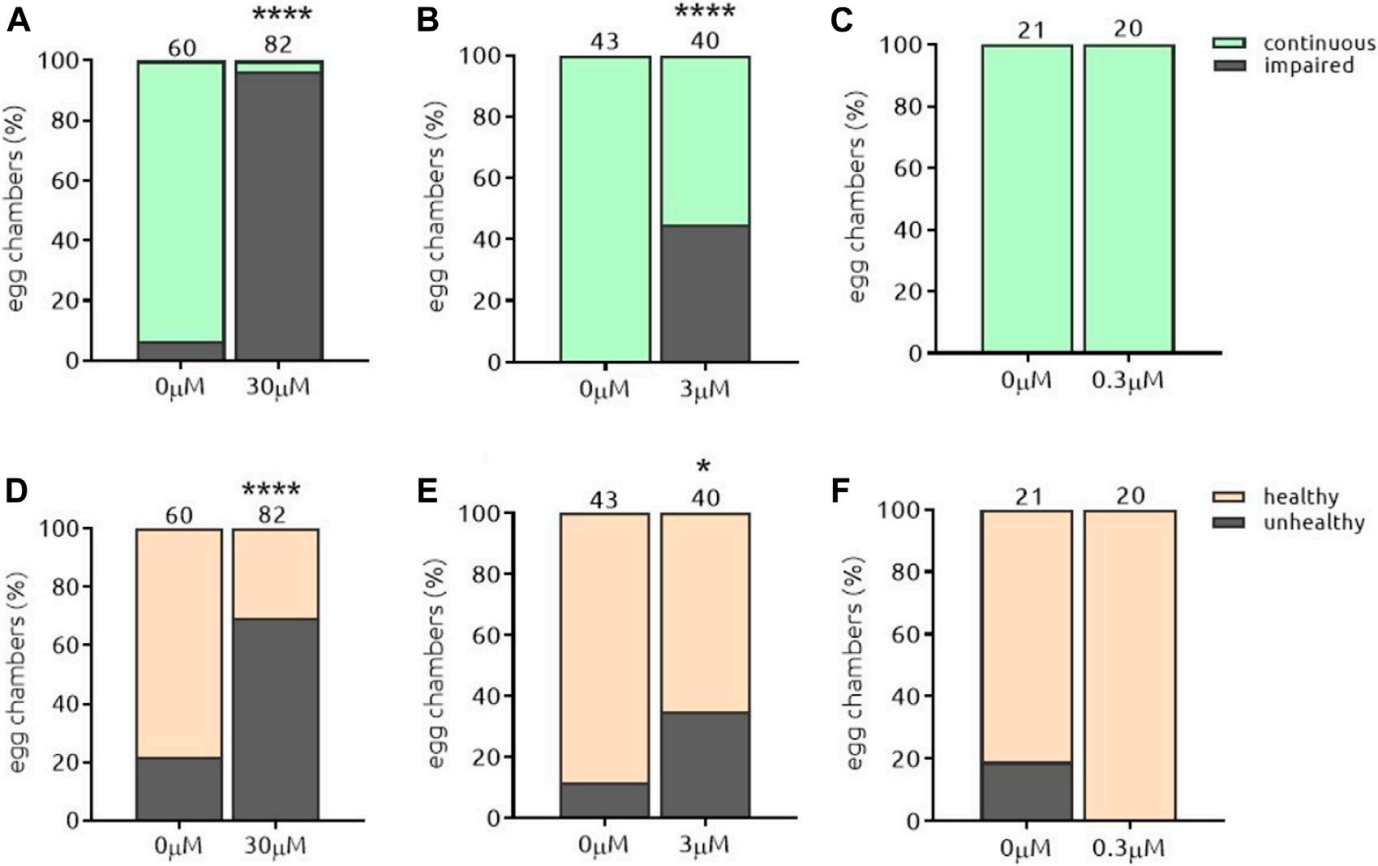
*the*-104b induces Par-6 ring impairment and toxicity in a dose-response manner. Dissected egg chambers were incubated with t*he-*104 b for 2 h at 0.3, three or 30 µM or DMSO only (0 µM) in 0.5 ml tubes for 2 h at 25°C in the dark and in the absence of movement (no shaking). Egg chambers were washed, fixed and transferred to glass slide for the detection of GFP signal using a fluorescent microscope for evaluation of Par-6 ring impairment **(A–C)**. Epithelial and nurse cell integrity was evaluated and considered unviable by identifying the presence of a “blebbing-like” phenotype **(D–E)**. Egg chambers containing at least one unviable cell were already considered unhealthy. *p < 0.05; ****p < 0.0001.

Amphotericin B (*the-104a*) and Nystatin (*the-104b*) are polyene antibiotics (toxic to fungi but not bacteria), both with a broad spectrum of antifungal activity. They were identified independently but named a and b as their chemical structures are similar and differ only in the position of hydroxyl residues in the hydrophilic side (Figure 6). *the-104a* impaired the Par-6 ring independently of the dose in the range tested (Figures 7A–C); egg chambers containing unhealthy cells were also found to be increased in number compared to the control at all doses tested (Figures 7D–F). Even at the lowest dose, 42% of egg chamber cells were unhealthy when incubated with *the-104a.* Incubation with the-*104b* induced Par-6 ring impairment in a dose-dependent manner (Figures 8A–C). The highest dose had a stronger effect on cell viability whereas incubation with 0.3 µM did not affect cellular health (Figures 8D–F). Despite the structural similarities between the compounds, the broadest window of specific activity was at different doses: 0.3 µM for *the-104a* and 3 µM for *the-104b* (Table 2). Due to their well-known toxicity (Laniado-Laborín and Cabrales-Vargas, 2009; Semis et al., 2013), these compounds were not listed for further development.

#### 5.3.2. The-105

**FIGURE 9:**
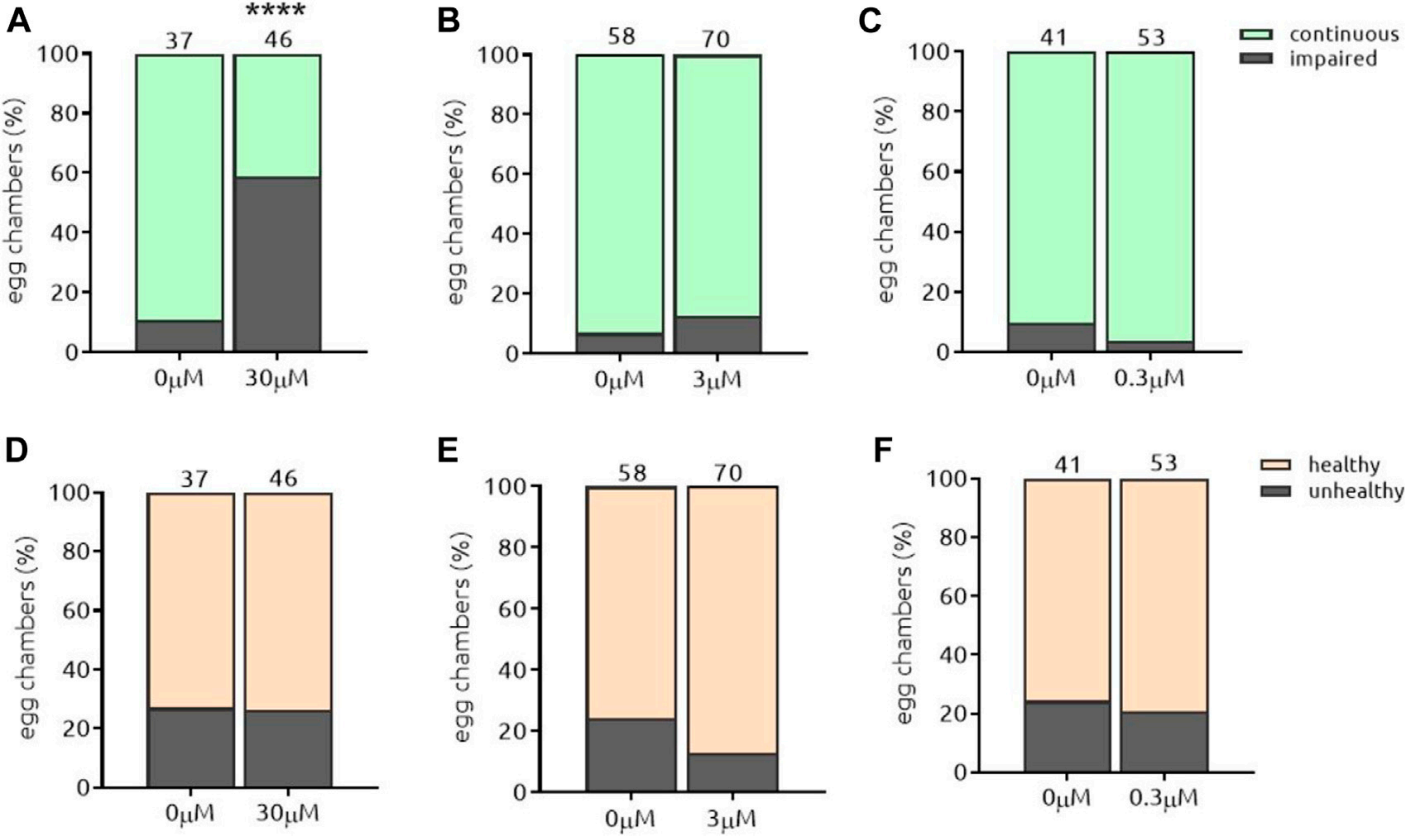
*the*-105 induces Par-6 ring impairment only at the highest dose and is not toxic to cells. Dissected egg chambers were incubated with t*he-*105 at three different concentrations (0.3, 3 and 30 µm) or DMSO only (0 µm) for 2 h at 25°C in 0.5 ml tubes in the dark without shaking. After incubation cells were washed, fixed with PFA and evaluated for Par-6 ring impairment **(A–C)**. Epithelial and nurse cell integrity was evaluated and considered unviable by identifying the presence of a “blebbing-like” phenotype **(D–E)**. Egg chambers containing at least one unviable cell were already considered unhealthy. ****p < 0.0001.

Auranofin (*the-105*) was the next compound to induce Par-6 ring impairment. Auranofin is a gold-containing molecule, prescribed for rheumatoid arthritis and is now under study as a potential treatment for cancers due to its inhibitory effect on PKC signaling, which is required to maintain tumor-initiating phenotype (Y. Wang et al., 2013) (Butler et al., 2015). In our assay *the-105* was capable of impairing Par-6 ring in 58% of the egg chambers treated with 30 µM (Figure 9A). Lower doses did not have an effect (Figures 9B,C). Regarding toxicity, *the-105* did not affect cell viability (Figures 9D–F). The broadest window of specific activity for this compound was 30 µM (Table 2). Interestingly, aurothiomalate (ATM)—the same chemical class molecule as auranofin—was demonstrated to disrupt the binding of PKCζ to Par-6 in pancreatic cancer cells (Butler et al., 2015). The blinded identification of this gold salt in our assay underpins the logic behind the assay, that disruption of aPKC/Par-6 complex can be associated with Par-6 mislocalization and a potential mechanism underlying Par-6 ring impairment. Due to known side effects of this compound (Chaffman et al., 1984), we ruled-out possibility of further development.

#### 5.3.3. The-110 and The-111

**FIGURE 10:**
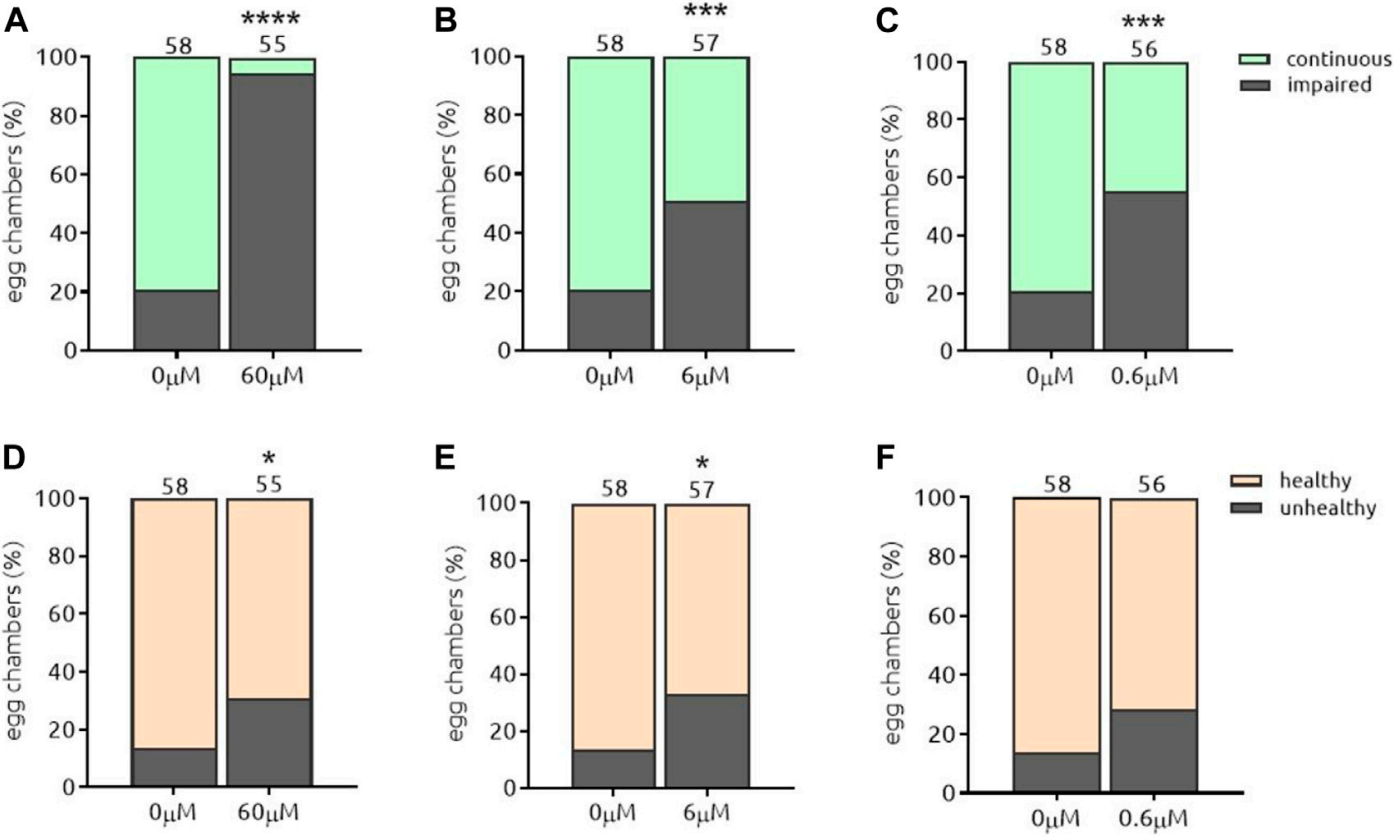
*the*-110 induces Par-6 ring impairment at all doses and is slightly toxic at the highest and intermediate doses. Dissected egg chambers were incubated with t*he-*110 at concentrations of 0.6, 6 and 60 μM, or DMSO only (0 µm) in 0.5 ml tubes for 2 h at 25°C in the dark, no shaking. Cells were then washed, fixed in PFA and evaluated for Par-6 ring impairment **(A–C)**. Epithelial and nurse cell integrity was evaluated and considered unviable by identifying the presence of a “blebbing-like” phenotype **(D–E)**. Egg chambers containing at least one unviable cell were already considered unhealthy. *p < 0.05; ***p < 0.001; ****p < 0.0001.

**FIGURE 11:**
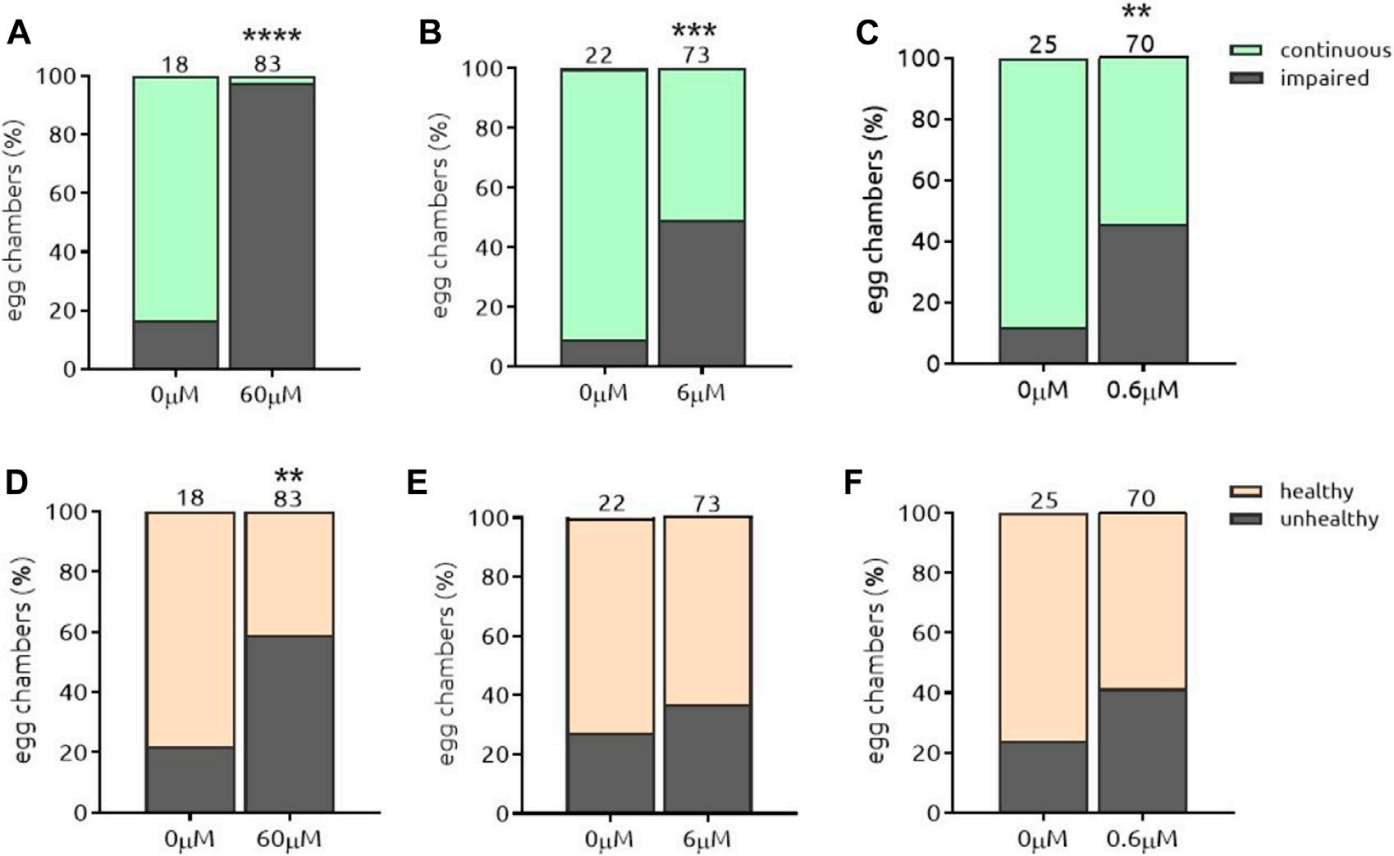
*the*-111 induces Par-6 ring impairment at all doses and is toxic at the highest dose. Dissected egg chambers were incubated with t*he-*111 at concentrations of 0.6, 6 and 60 μM, or DMSO only (0 µm) in 0.5 ml tubes for 2 h at 25°C in the dark, no shaking. Cells were then washed, fixed in PFA and evaluated for Par-6 ring impairment **(A–C)**. Epithelial and nurse cell integrity was evaluated and considered unviable by identifying the presence of a “blebbing-like” phenotype **(D–E)**. Egg chambers containing at least one unviable cell were already considered unhealthy. **p < 0.001; ***p < 0.001; ****p < 0.0001.

Myricetin (*the-110*) and quercetin (*the-111*) are plant-derived flavonoids present in fruits, vegetables, grains, leaves, tea. They are structurally related and well known for both anti-oxidant and anti-inflammatory activities (Semwal et al., 2016; Xu et al., 2019; Mlcek et al., 2016). Both compounds induced a strong effect on Par-6 modulation, most pronounced at the highest dose (60 µM) (Figures 10A, 11A) which led to Par-6 ring impairment in 96 and 97% of egg chambers for *the-110* and *the-111*, respectively. Reducing 10-fold the amount of either compound also decreased the percentage of egg chambers with Par-6 ring impairment (Figure 10B, 11B). Approximately 50% of egg chambers with ring impairment was also observed when the lowest dose of *the-110* and *the-111* were applied (Figure 10C and Figure 11C). Cells in egg chambers were slightly more sensitive to doses of 60 and 6 µM of *the-110* (Figures 10D,E); a lower viability (59% of egg chambers affected) was also detected when egg chambers were incubated with *the-111* at 60 µM (Figure 11D) but not the other doses (Figures 11E–F). The broadest window of specific activity was 60 and 6 µM for *the-110* and *the-111*, respectively (Table 2). Although in our assays, the maximum dose of quercetin (60 µM) suggested toxicity, the use of high-purity quercetin as food ingredient to 1000 mg daily is considered safe (Andres et al., 2018). Both compounds were selected for validation of their barrier function properties.

### *The-110* and *The-111* Increase the Epithelial Barrier Strength

**FIGURE 12:**
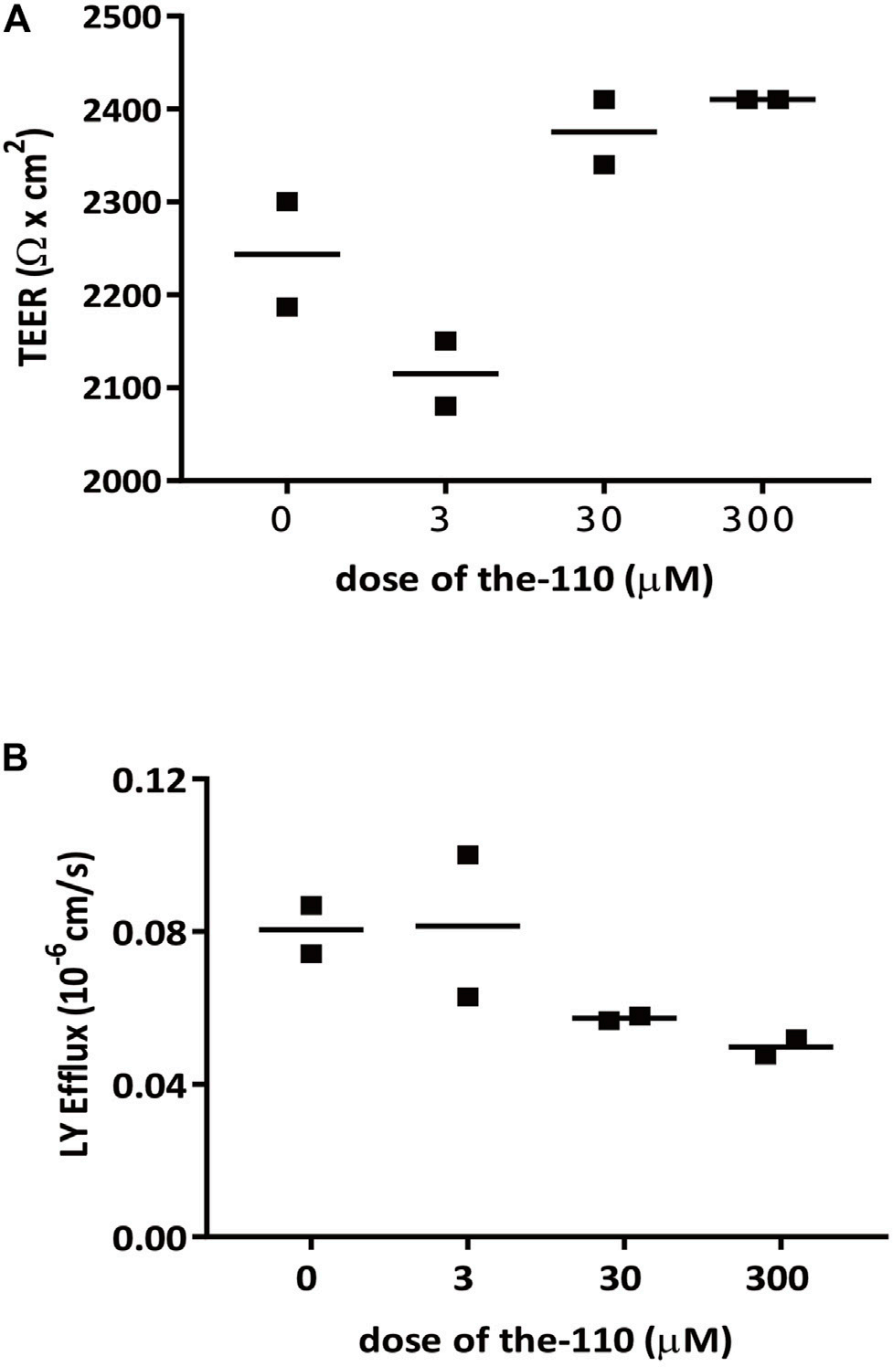
*the*-110 increases TEER and reduces paracellular permeability in Caco-2 epithelial cells. Assay was performed by Cyprotex according to their established protocol. Briefly, caco-2 cells from passage 40–60 were seeded in duplicate in a transwell insert until confluent. Cells were treated with myricetin at three different concentrations (0.3, 3 and 30 µM) or DMSO only (0 µM), in the presence of Lucifer Yellow (LY) for 2 h. Permeability was measured by transepithelial electrical resistance (TEER) and LY efflux across the barrier.

**FIGURE 13:**
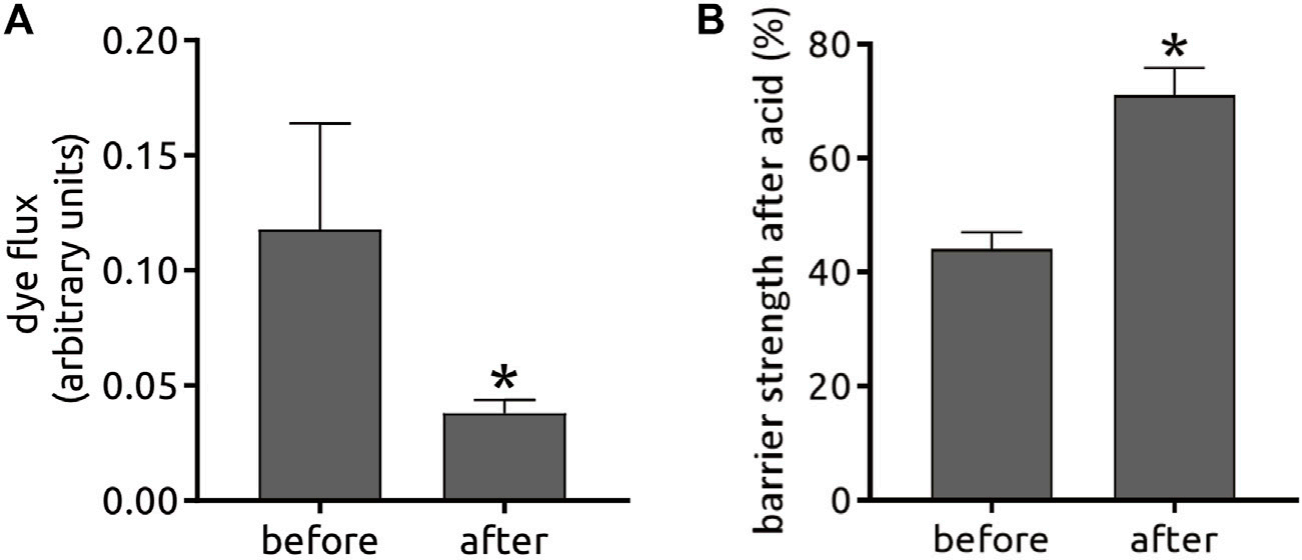
An independent open label clinical trial with quercetin was conducted in GERD patients under eligibility criteria such as age, GERD diagnosis, abnormal 24 h-pH monitoring or past responsiveness to PPI therapy (clinicaltrials.gov NCT02226484) among other criteria described in the Methods. Patients took 500 mg quercetin orally twice-a-day for 6 weeks and were monitored 1 week before and during the duration of the trial. After 6 weeks, biopsies of the esophagus were taken to determine changes in the barrier function by dye flux **(A)** and resistance to exposure to hydrochloric (gastric) acid **(B)**. These results are unpublished and the use of information is disclosed under a non-exclusive license agreement between the University of North Carolina at Chapell Hill and Epinutra (Thelial BV).

In mammalian epithelial cells, overexpression of Par-6 has been shown to delay the assembly of tight junctions (Joberty et al., 2000; Gao et al., 2002). Since our compounds induced Par-6 modulation we decided to test if myricetin (*the-110*) could increase epithelial barrier strength using human colorectal adenocarcinoma cell line (Caco-2)—a widely used model for permeability assays. Experiments were performed by Cyprotex (www.cyprotex.com). Incubation with *the-110* for 2 h lead to a slight increase in transepithelial electrical resistance (TEER) in 5.8 and 7.4% for the doses of 30 and 300 μM, respectively compared to the control (Figure 12A). Barrier strength was also measured by lucifer yellow dye efflux; 30 and 300 µM showed an effect of 29 and 37.5%, respectively, compared to the control, on reducing the passage of the dye through the cell monolayer (Figure 12B). Regarding quercetin (*the-111*), an independent research group at The University of North Carolina at Chapell Hill had identified quercetin as a candidate in their pre-clinical research; based on this convergent finding, a clinical trial was conducted by them in gastroesophageal reflux disease gastroesophageal reflux disease (GERD) sufferers (Clinicaltrials.gov NCT02226484). This trial revealed that oral intake of quercetin twice-a-day for 6 weeks significantly strengthened the barrier function of the esophageal epithelium and specifically it improved epithelial resistance to damage upon exposure to hydrochloric (gastric) acid (unpublished) (Figure 13). As the results from the clinical trial are so far unpublished, the disclosure is limited in order not to impinge on the novelty of the full data-set. Taken together, these results reinforce our hypothesis that Par-6 ring impairment is suitable read-out for screening of compounds that could play a role in reinforcement of epithelial barriers.

## Discussion

We here describe screening platform *the*LITE™ to find Par-6 targeted compounds based on live epithelial tissue from *Drosophila* egg chambers. We found 10 hits in a total of 2,400 screened compounds giving a hit frequency in the range of 0.4%. We discuss six of them: *the103*, *the104a*, *the-104b*, *the-105*, *the-110* and *the-111*. The other four hits were excluded from further study because they did not match pre-determined criteria (toxicity, nature of the compound, etc). Food constituents myricetin (*the-110*) and quercetin (*the-111*) were validated for their capacity in increasing epithelial barrier resistance *in vitro* (myricetin) and via a clinical trial (quercetin).

*Drosophila* and human genomes share approximately 80–90% in conserved functional domains: *Drosophila* represent a valid alternative in the drug discovery process, with various examples of success (Pandey and Nichols 2011). With *the*LITE™ we respond to a specific need in candidate compound discovery: providing whole tissue data early on in the discovery process. Candidate compounds require subsequent testing in models, but generating high quality *in vivo* data at an early stage can reduce costly later stage attrition.

Egg chambers were evaluated for continuous or impaired Par-6-GFP ring and cell viability was based on “blebbing” phenotype. Clearly, the fact that toxicity could result in *prima face* loss of Par-6 signal is a potential weakness of this approach; for this reason, great emphasis was put on scoring toxicity readouts to ensure we were measuring a real and robust window of specific activity. We established the broadest window of specific activity for each compound, corresponding to the highest number of egg chambers that had both viable cells and Par-6 ring impairment. Candidates were numbered sequentially from *the-103* onwards also to illustrate how we gradually built confidence in the screen, finally finding candidate compounds which match all pre-determined requirements for further development. Antimycin A (*the-103*) was the first compound which hit the predefined screening criteria of Par-6 ring impairment without overt toxicity to the egg chambers apart form the highest dose. Amphotericin B (*the-104a*) and Nystatin (*the-104b*) were identified separately; their know structural and functional similarity underlines the specificity of the assay. Identification of *the-105* pinpointed that our technology can pick-up the specific activity we were looking for, as it is reported that aurothiomalate (ATM), which is from the same chemical class molecule as auranofin (gold-containing molecule), inhibited Par6:PKCζ interaction in assays with pancreatic cancer cells (Butler et al., 2015). None of these compounds however matched requirements for further development due to their known toxic properties or side effects. Antimycin A is a mitochondrial toxin and has been shown to induce cell death in human retinal epithelium in a dose- and time-dependent manner (Hytti et al., 2019). Amphotericin B (*the-104a*) and Nystatin (*the-104b*) are structurally closely related polyene antibiotics that are used as antifungal agents; they bind to sterol on the plasma membrane of fungi, forming pores, causing leakage and eventually death. Nystatin is restricted in clinical usage to topical or oral infections because there is no formulation suitable for systemic treatment; Amphotericin B is used to treat systemic infections but produces various acute and chronic side effects. Nephrotoxicity is the most common, caused by Amphotericin B altering tubular cell membrane permeability (Sawaya et al., 1995). Auranofin (*the-105*) is an orally active gold compound to treat rheumatoid arthritis. It is relatively well tolerated in most patients, but gastrointestinal reactions can be common adverse effects (Chaffman et al., 1984).

We then identified two compounds which met the metric for further development: *the-110* (myricetin) and *the-111* (quercetin). These flavonoids are present in berries, vegetables, teas, fruits and grains (quercetin levels are especially high in apples and onions); both are structurally related and are mainly present in glycosidically-bound form (Andres et al., 2018; Taheri et al., 2020). Myricetin and quercetin have been widely studied and positive biological properties such as anti-oxidant, anti-inflammatory, anti-allergenic, anti-cancer, anti-diabetisc recorded (Li et al., 2016; Semwal et al., 2016; Xu et al., 2019; Taheri et al., 2020). Clearly there is a broad spectrum of activities but relevant here are the effects of these flavonoids in tight junction regulation (Suzuki and Hara 2011). Myricetin has been shown to enhance—in a dose and time-dependent manner—barrier function in rat (IEC-6) intestinal epithelial cell lines (Fan et al., 2020). When tested in Caco2 cell monolayers, myricetin had no effect on TEER and a slight but statistically significant dose-dependent effect in LY efflux (Suzuki and Hara 2009). Our observations with Caco_2_ cell monolayers in the presence of myricetin (performed by Cyprotex) also showed a slight increase in TEER and decrease in LY efflux already at 2 h after treatment. The studies listed above (Suzuki and Hara 2009; Fan et al., 2020) also evaluated the effect of quercetin in increasing barrier strength; compared to myricetin, quercetin had a higher barrier-promoting efficiency in both Caco_2_ and IEC-6 cells. The dynamics of TEER in the presence of quercetin may be complex; in canine kidney epithelial cell line (Madin-Darby Canine kidney cells (MDCK-II)) there were two waves: TEER increased 3–5 h, decreased at 18 h and increased at 36–48 h reaching stability (Gamero-Estevez et al., 2019).

Studies have associated quercetin-induced epithelial barrier resistance with changes in the protein expression and localization of claudin family members. A decrease in claudin-2 and increase in claudin-3 and -4 were observed during increase of TEER in MDCK-II cells treated with quercetin (Gamero-Estevez et al., 2019). Other studies have also found a correlation between higher epithelial barrier function and increased claudin-4 expression after quercetin exposure in other models (Amasheh et al., 2008; Suzuki and Hara 2009; Mercado et al., 2013). Suzuki and Hara (2009) also showed a biphasic behavior of TEER in Caco2 cell monolayers incubated with quercetin for 48 h; in the early phase quercetin induced the assembly of ZO-2, claudin-1 and occludin and at a later phase (after 12 h), claudin-4 had an additional role in maintaining increased barrier function. In rat intestinal epithelial cells (IEC-6), mRNA expression of ZO-1, claudin-1 and occludin were upregulated at 24 h after quercetin exposure (Fan et al., 2020).

The molecular mechanisms underlying quercetin-induced tight junction assembly have been studied. Amasheh et al. (2008) showed that quercetin activates the claudin-4 promoter and stimulates claudin-4 transcription, leading to higher amounts of claudin-4 being assembled into the strands of tight junctions. Studies by Suzuki and Hara (2009) demonstrate that quercetin inhibited PKCσ activity on Ser643, which strongly suggests that the suppression of the activity of this kinase leads to phosphorylation of occludin and promotion of tight junction assembly. Other reports point to an effect of quercetin in activating AMP-activated protein kinase (AMPK) (Jiang et al., 2016; Qiu et al., 2018) which has been shown to play a key role in both assembly and stability of apical junctions via the phosphorylation of TJ proteins and associated proteins (Rowart et al., 2018; Olivier et al., 2019).

We have not investigated in full detail the manner of how our tested compounds affect Par-6 ring impairment and here speculate on potential mechanisms based on published data. In the case of *the-103*, Par-6 ring impairment could be an indirect effect of mislocalization of the cytoskeletal protein lethal giant larvae (Lgl), which is required for Par-6 maintenance (Hutterer et al., 2004). Our hypothesis is based on the observation that in *D. melanogaster* embryonic epithelial cells, antimycin (*the-103*) induced diffusion of Lgl from the plasma membrane to the cytosol (Dong et al., 2015).

In the cases of amphotericin B (*the-104*) and auranofin (*the-105*) we speculate that Par-6 ring impairment could be a consequence of the disruption of the complex aPKC: Par-6 and so, allowing Par-6 to diffuse in the cytosol. Macrophages treated with amphotericin B (*the-104a*) showed a decrease in protein expression of PKCζ (Mukherjee et al., 2010); in pancreatic cancer cells, treatment with ATM, which is a molecule in the same chemical class as auranofin (*the-105*), disrupted the binding of either PKCι or PKCζ to Par-6 (Butler et al., 2015). It has been shown that stabilization of Par-6 protein levels depends on its co-expression with aPKC meaning that aPKC expression increases Par-6 steady-state levels (Gunaratne et al., 2013). This is aligned with the recent report that in *Drosphila* photoreceptors, binding of aPKC to Par-6 is required for the apical localization of Par-6 and accumulation of the Par-6-aPKC complex (Nunes de Almeida et al., 2019).

The effect of myricetin in Par-6 ring impairment may be linked to E-cadherin. It has been recently shown that myricetin inhibited EMT in hepatocellular cancer cell line MHCC97H by increasing E-cadherin and decreasing N-cadherin and vimentin mRNA expression levels (Ma et al., 2019). E-cadherin is a major constituent of adherens junctions and plays an important role in cell-cell adhesion. Reduced surface expression of E-cadherin following IFN-γ treatment in human colonic epithelial cell line destabilized the epithelial monolayer (Smyth et al., 2012). Combining these two observations, we formulate a hypothesis for Par-6 redistribution leveraged by the E-cadherin pathway: Since E-cadherin can physically interact with Baz/Par-3 (Achilleos et al., 2010) and Baz/Par-3 colocalizes with Par-6-aPKC, increased expression of E-cadherin could impact Par6 via a disturbance in Baz/Par-3 due to increase in the expression of E-cadherin.

In photoreceptors of *Drosophila* the correct apical localization of Par-6 depends on the binding of Par-6 to Cdc42 (Nunes de Almeida et al., 2019). Interestingly, Cdc42 is a target for quercetin; comparative molecular docking studies show that quercetin has binding affinity for Cdc42 (Amanzadeh et al., 2019). We speculate that Par-6 modulation after quercetin treatment could be a consequence of the interaction between Cdc42 and the flavonoid which could in turn impact the accumulation of the Par-6 at the apical membrane.

Finally, it is important to highlight that in our assays, Par-6 ring impairment is a readout and does not necessarily imply that the compound that induced Par-6 ring modulation is a candidate for regulation of epithelial barrier integrity/reinforcement. Evaluating its capacity to increase cell-cell contact, preferentially by regulating tight junction is the next step. We focused on the two flavonoids (*the*-110, -111) because they are well-known food ingredients with excellent human safety profiles and their biological properties have been widely studied. Our results with myricetin on epithelial resistance of Caco2 cell monolayers (a standard permeability assay performed by Cyprotex Ltd.) suggested myricetin as a potential candidate. The sheer amount and quality of data backing quercetin means this molecule is our candidate of choice. A proprietary formulation of quercetin targeting esophagus barrier strength is under clinical evaluation and being commercialized.

## Data Availability

The original contributions presented in the study are included in the article/Supplementary Material, further inquiries can be directed to the corresponding author.
