# Supplementary figures and images for: theLiTE™: A Screening Platform to Identify Compounds that Reinforce Tight Junctions
Source: Front Pharmacol. 2022 Jan 4;12:752787. doi: 10.3389/fphar.2021.752787 (PMC8771259; doi:10.3389/fphar.2021.752787)

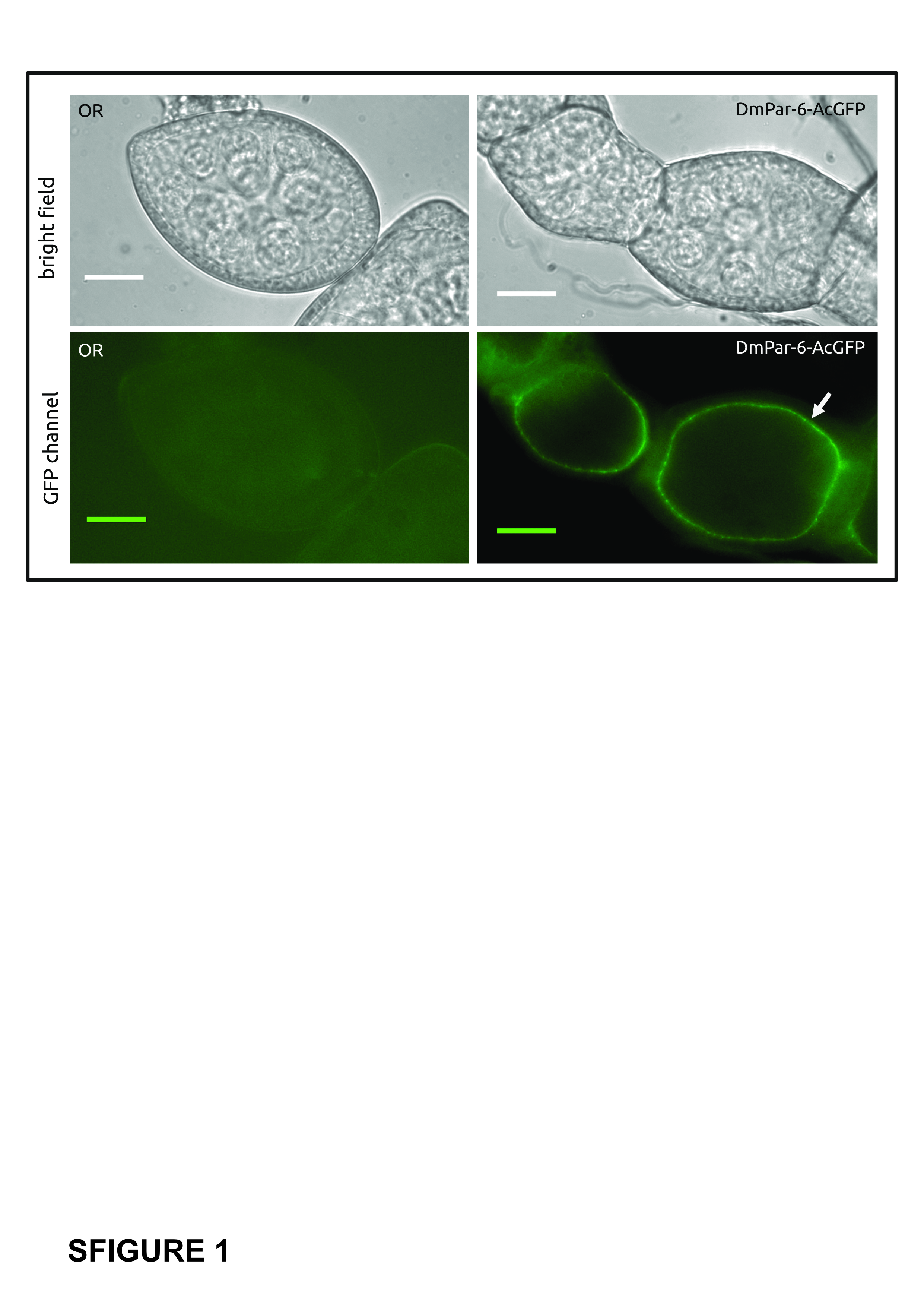

Supplement: Supplementary file 1 [file Image1.JPEG]

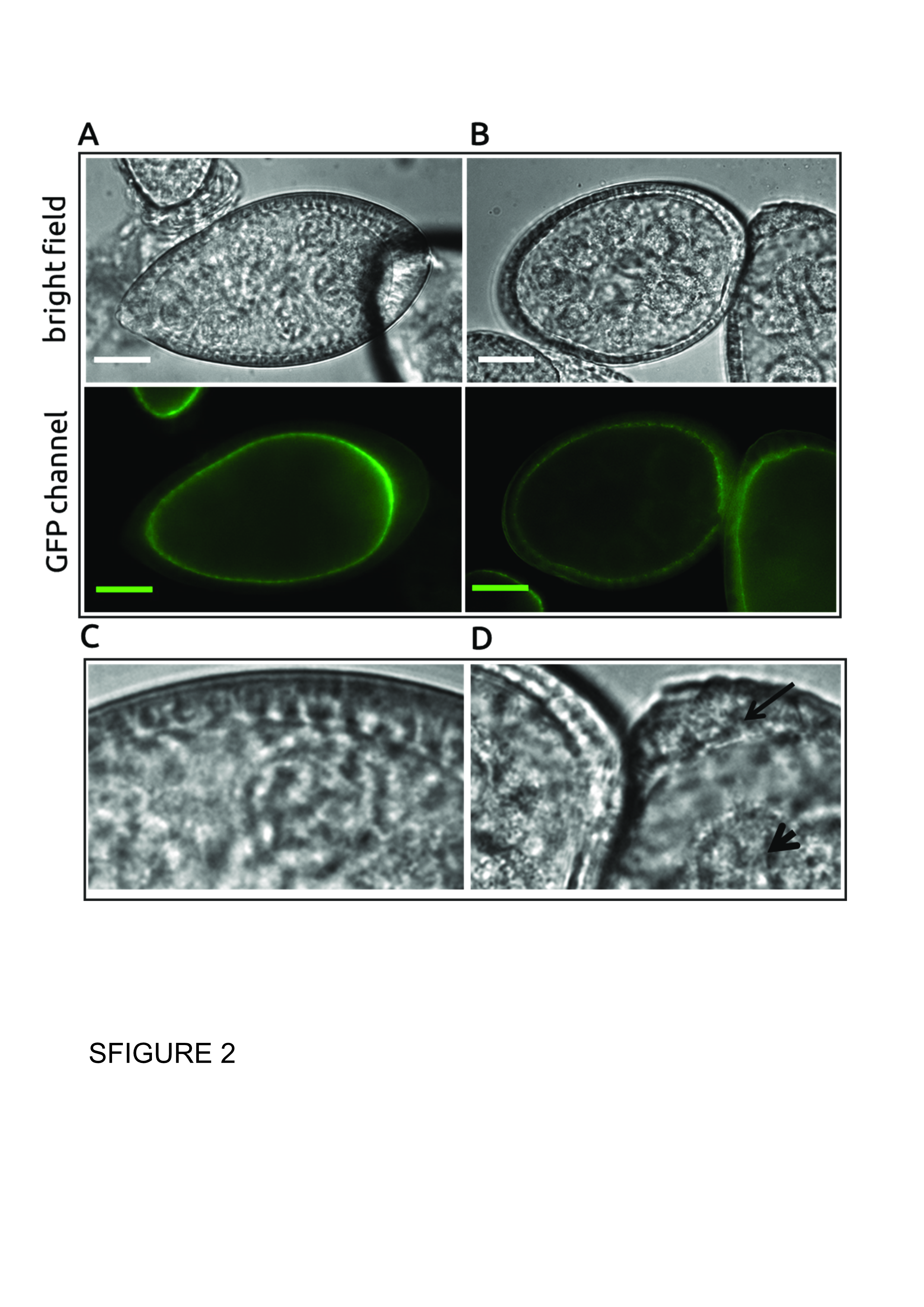

Supplement: Supplementary file 2 [file Image2.JPEG]
