# Supplementary material for: theLiTE™: A Screening Platform to Identify Compounds that Reinforce Tight Junctions
Source: Front Pharmacol. 2022 Jan 4;12:752787. doi: 10.3389/fphar.2021.752787 (PMC8771259; doi:10.3389/fphar.2021.752787)
Supplement: Supplementary file 3 [file DataSheet1.docx]

**Table 1.** List of the compounds that induced Par-6 ring impairment.

| Compound Name (CAS#) | Compound Code |
| --- | --- |
| Antimycin A | the-103 |
| Amphoterracin B | the-104a |
| Nystatin | the-104b |
| Auranofin | the-105 |
| Myricetin | the-110 |
| Quercetin | the-111 |

**Table 2.** Par-6 ring impairment in egg chambers containing healthy cells after incubation with compound. Highlighted is the dose within the window of specific activity.

|  |  | **% of egg chambers containing** | |
| --- | --- | --- | --- |
| **Compound** | **Dose (µM)** | Par-6 ring impairment | Par-6 ring impairment and healthy cells |
|  | 0.0 | 16.5 | 94.7 |
| **the-103** | 0.3 | 100 **** | 57.8 |
|  | 3.0 | 73.7**** | 76.3 |
|  | 30.0 | 100**** | 8.9 |
|  | 0.0 | 15.4 | 94.2 |
| **the-104a** | 0.3 | 70.8*** | 58.4 |
|  | 3.0 | 100**** | 45.9 |
|  | 30.0 | 96**** | 22 |
|  | 0.0 | 6.6 | 95 |
| **the-104b** | 0.3 | 0 | 100 |
|  | 3.0 | 45*** | 70 |
|  | 30.0 | 96.3**** | 31.7 |
|  | 0.0 | 6.9 | 94.8 |
| **the-105** | 0.3 | 3.8 | 98.1 |
|  | 3.0 | 12.8 | 95.7 |
|  | 30.0 | 58.7**** | 78.2 |
|  | 0.0 | 20.7 | 93.1 |
| **the-110** | 0.6 | 55.3*** | 75 |
|  | 6.0 | 50.8*** | 80.7 |
|  | 60.0 | 94.5**** | 76.3 |
|  | 0.0 | 12.3 | 98.5 |
| **the-111** | 0.6 | 45.7** | 71.4 |
|  | 6.0 | 49.3*** | 76.7 |
|  | 60.0 | 97.6**** | 40.9 |
